# Setting priorities for mental health care in Nepal: a formative study

**DOI:** 10.1186/1471-244X-13-332

**Published:** 2013-12-05

**Authors:** Mark JD Jordans, Nagendra P Luitel, Mark Tomlinson, Ivan H Komproe

**Affiliations:** 1 Research and Development, HealthNet TPO, Amsterdam, The Netherlands; 2 Center for Global Mental Health, King’s College London, London, UK; 3 Research Department, Transcultural Psychosocial Organization (TPO), Kathmandu, Nepal; 4 Psychology Department, Stellenbosch University, Stellenbosch, South Africa; 5 Center for Public Mental Health, University of Cape Town, Cape Town, South Africa; 6 Faculty of Social and Behavioural Sciences, Utrecht University, Utrecht, The Netherlands

**Keywords:** Nepal, Mental health care packages, Formative research, Development

## Abstract

**Background:**

There is an urgent need to address the massive treatment gap for mental health problems, especially in low income settings. Packages of care integrated in routine primary health care are posited as a strategy to scale-up mental health care, yet more needs to be known about the most feasible and effective way to go about this.

**Methods:**

The study follows a combined methods design that includes engaging an expert panel in a priority setting exercise, running workshops to develop a Theory of Change and conducting in-depth qualitative interviews and focus group discussions with key stakeholders. The results of each research step were taken forward to inform the subsequent one.

**Results:**

There was strong endorsement for a system of care that encompasses both the perspectives of health facility and the community. Issues related to increasing access and demand, guaranteeing a sustainable supply of psychotropic medicine, adequate human resourcing, and ensuring positive family involvement came up as priority areas of attention.

**Conclusion:**

The study underlines many of the known barriers in developing mental health services. At the same time it provides a distinct pathway and concrete recommendations for overcoming these challenges in Nepal.

## Background

Mental health needs of people in low and middle income countries are largely unmet, with only a small fraction of those affected receiving adequate treatment [1,2]. The ambition of providing mental health care is beset by multiple challenges, which include limited mental health specialists and available treatments, often due to a lack of policies and financial resources [3]. There is an urgent need to identify effective strategies that overcome such barriers in the delivering interventions. The lack of this evidence is a major challenge in the process of scaling up mental health care in Low- and Middle Income Countries [4].

Multiple studies have demonstrated that interventions provided by trained non-specialists (i.e. task shifting) in low income settings can be effective in achieving significant treatment gain [5-7]. However, many of these are proof-of-concept studies as they remain relatively small-scale and treatment-specific. There is also a need for the implementation of packages of care that combine evidence-based treatments for multiple mental disorders rather than stand-alone interventions for single disorders [8]. To make significant progress in reducing the treatment gap, a comprehensive and multi-tiered approach is needed to bring evidence-based treatments to a national-level scale. With that aim, the World Health Organization has launched its mental health Gap Action Program (mhGAP) [9] which promotes the integration of mental health into primary health care.

The goal of developing population-wide mental health services in LMIC is not new. Previous programs have shown that practice-oriented mental health trainings for general health workers led to substantially increased uptake of mental health care services in Afghanistan [10]; while task-shifting has been shown to substantially reduce the human resource needs to address the treatment gap at minimal costs in South Africa [11]; and a large-scale mental health awareness program markedly increased referral to services in Nigeria [12]. At the same time, studies have demonstrated that training alone did little to improve the management of mental health problems [13]. Also in Nepal, there has been significant efforts made towards the development of a community mental health delivery program integrated in the public health system, yet without it being brought to scale [14]. The major challenge now is to know how best to address specific health systems constraints (including human resources, capacity building, information systems, health financing and service delivery), when trying to horizontally integrate mental health into primary care [15], given that there is no single best practice model available [16]. Consequently, there is an urgent need to better understand how a comprehensive mental health approach, encompassing services within primary health care and the surrounding community, can be developed and implemented.

The PRogramme for Improvement of Mental health carE (PRIME), a research consortium working in India, Uganda, Ethiopia, South Africa and Nepal, aims to evaluate the feasibility, acceptability and impact of a multi-faceted mental health care approach that targets the health facility, community and health service organization [17]. Each site, in close partnership with Ministries of Health, will develop, implement, evaluate and scale-up a comprehensive mental health care plan.

While the development of a scalable model of mental health care is challenging in any LMIC, in fragile states or complex humanitarian emergencies this is particularly the case due to the increased risk of mental health problems [18,19], and poor pre-existing health systems [20]. The present study is part of the formative phase of PRIME, which concentrates on assessing the priorities, processes and building blocks of developing such mental health care plan. The aim of the current study is to investigate the challenges and opportunities for the development and fine-tuning of a comprehensive mental health care plan in post-conflict Nepal. The study follows a combined methods design that includes a priority setting study, running workshops to develop a Theory of Change and conducting a qualitative study.

## Methods

### Setting

The research was conducted in Chitwan, a district in southern Nepal. Nepal is a low income country, one of the poorest countries in Asia and is categorized by the World Bank as a fragile state [21]. The country is passing through a transition following a 10-year intra-state conflict, between government forces and Maoists insurgents, which raged between 1996 and 2006 and claimed more than 13,000 lives. As the conflict was rooted in an egalitarian ideology among disadvantaged rural populations against the ruling elite in the capital city, rural districts such as Chitwan were heavily impacted. Previous studies have demonstrated the impact of political violence on psychosocial wellbeing and mental health in Nepal [22]. Furthermore, the conflict has further shattered an already weak health care system. A recent prospective study showed that conflict exposure predicted increases in anxiety whereas socio-economic factors and non-conflict stressful life events were the major predictors of depression [18]. It is against the backdrop of recent violence and ongoing poverty that the PRIME program takes place in Nepal.

### Sample and procedure

The study consisted of three stages, each with a separate methodology. First, a priority setting exercise was conducted among an expert panel of mental health experts in Nepal to determine the most urgent mental health problems to target in the future mental health care plan. An initial list of senior mental health professionals (n = 21) was drafted. Inclusion was based on known track record (more than 5 years of experience in clinical services) and held positions (heads of psychiatry departments in Nepal’s universities and hospitals). All approached participants were asked to name other experts to be added (n = 9). The final group of experts was invited to participate in the study of whom 26 participated (86.6% response rate), including psychiatrists (58%), psychologists (31%), psychosocial counselors (8%) and a psychiatric nurse (3%), with a mean of 19.1 years (SD = 9.9) of professional experience. Among the non-responders one refused, one was not available and two did not reply after two reminders. For the expert panel we used a questionnaire that asked each participant to prioritize among all mental disorders that are included in the mhGAP. Scoring was done following three criteria (0, 0.5 or 1, for increasing importance or agreement, and blank for no opinion), commonness of the disorder in their practice, relevance of the disorder within the cultural context of Nepal, and perceived feasibility to provide treatment within non-specialist settings.

Second, Theory of Change (TOC) workshops were organized with primary health care staff and policy makers to ascertain the different intermediate outcomes that constitute the expected pathway to change. Change here refers to the implementation of an integrated mental health plan leading to improved functioning among people with mental health problems. The TOC workshops followed a procedure as proposed by Anderson [23], which entails asking participants to map a causal chain of pre-conditions (or preliminary outcomes), assumptions and interventions leading to an ultimate outcome. In our study we conducted four workshops; a first 1-day workshop with mainly health care providers to identify pre-conditions; a second ½-day workshop with mainly policy makers to discuss and adapt the results of the first workshop; a third 1-day workshop with the initial group to include indicators for the developed TOC and discuss disorder specific adaptations, and a final ½-day workshop with the same policy makers to finalize the TOC that came out of the third workshop. The TOC workshops consisted of 6 mental health professionals (psychiatrists, psychologists and psychiatric nurse), 11 primary health care staff, 7 policy makers or health managers, 2 representatives of mental health organizations, and 1 representative of a mental health user group. Participants were included following convenience sampling, aiming for representation of the different stakeholder groups. Of the total sample of 27, 15 (55.6%) were part of both cycles of workshops, the others were part of only the first or second.

Third, an explorative qualitative study using Focus Group Discussions (FGD) and semi-structured Key Informant Interviews (KII) was conducted to assess implementation issues for each of the TOC building blocks. The research question was; what are the opinions and perceptions of community member, health workers and policy maker on provision of mental health care at the community level and making it more widely available? The core interview templates were developed by the PRIME consortium, subsequently adapted for Nepal. The adaptation consisted of adding questions related to feasibility of the components of the TOC. Translation of the instruments was conducted following a systematic procedure developed for use in transcultural research that involves translation, back-translation and focus groups [24]. In total, 33 KII interviews and 9 FGDs were conducted. A total of 84 respondents participated in FGDs with average of 9 respondents in each group (group size ranging between 8 and 13). Consistent with the PRIME framework [17] and to ensure diversity of opinions of key stakeholders, the sample was selected to represent different levels (health organization, health facility and community), using purposive sampling technique done by research assistants, while snowball sampling was also used at the community level. At the health organization level both national and district level respondents were represented. At the health facility level, health units were selected to have a birthing facility and doctor or health assistant, and represent different geographical areas. Each respondent was recruited through a home visit or (in case of PHC workers and policy makers) a work place visit.

All formative research was conducted between April 2011 and November 2012. Ethical clearance for the study was gained from the Nepal Health Research Council and the Human Research Ethics Committee at the University of Cape Town. All interviews and workshops were conducted by a team of four local research assistants, who received a three-week training in basic research principles and skills. Informed consent was obtained from all participants to the study, for the qualitative study consent was in written form, which included assurance of confidentiality. See Table 1 for an overview of the sample.

**Table 1:** Respondents

	**Step 1: Priority setting**	**Step 2: TOC workshops**	**Step 3: Qualitative study**
Respondents	Capital n = 20	Service providers n = 19	FGD n = 84
	Periphery n = 6	Policy makers and Service users n = 8	KII n = 33
Gender			
Female	4 (15.4%)	9 (33.3%)	71 (60.7%)
Male	22 (84.6%)	18 (66.6%)	46 (39.3%)
Age (mean)	N/a	N/a	42.0
Total	26	27	117

*Note:* FGD Focus Group Discussion; KII Key Informant Interview; N/a Not available.

### Analyses

The results of the expert panel were analyzed by computing intermediate criterion-based scores by adding up all the informed (i.e., non-blank) answers (0, 0.5 or 1). The sum was divided by the number of received informed answers. Blanks were left out of the calculation in both numerator and denominator [25]. An overall priority score per disorder was calculated by taking the mean of the three criterion-based intermediate scores (no weighing of criteria was followed). The overall priority score ranges between 0 and 1.0 representing the level of collective agreement by experts per disorder.

The content and pathways as proposed by participants of the workshops were immediately displayed visually, thereby building up the TOC. Proposed changes were discussed and altered in the displayed TOC. All discussions and outputs were documented by a note taker, following a pre-established format.

Qualitative data from the explorative study was analyzed following a Framework Analyses approach [26]. First, a preliminary coding framework was developed based on a priori categories and new themes that emerged during initial reading of the data were added. This preliminary coding framework was fine-tuned by the research team based on a random selection (10%) of interviews. Final coding frameworks were subsequently applied to the entire data set, with sections of data reviewed by two researchers to evaluate comparability in coding. Analyses were done in NVIVO 9.0. All researchers involved in data collection or analyses had no other role in the program. Data analysis was performed on transcribed audio-taped interviews translated into English.

## Results

Table 2 presents the results of the priority setting exercise amongst the expert panel. The level of collective agreement for the different disorders ranged from .41 to .85. The disorders with highest ranking scores were depressive disorder, alcohol use disorder, epilepsy, anxiety disorder and psychoses – all of which had a total priority score of .75 and higher. The other disorders all had a markedly lower score on perceived treatment feasibility. In addition, the four disorders with the lowest collective agreement – developmental disorders, PTSD, behavioral disorders and dementia – also had low scores (.50 and lower) on cultural relevance.

**Table 2:** Results expert panel

**Disorder**	**Cultural relevance**	**Frequency**	**Feasibility**	**Total priority score**
Depression	.81	.98	.77	.85
Adolescent depression	.88	.92	.71	.83
Alcohol use disorder	.86	.96	.70	.84
Epilepsy	.83	.88	.79	.83
Anxiety	.77	.90	.75	.81
Psychoses	1.0	.83	.54	.79
Medically unexplained complaints	.71	.82	.68	.74
Bipolar disorder	.89	.87	.43	.73
Drugs	.82	.90	.38	.70
Behavioral problems	.50	.70	.39	.53
Developmental disorder	.60	.61	.31	.51
PTSD	.50	.61	.36	.49
Dementia	.46	.48	.29	.41

*Note*: PTSD Post Traumatic Stress Disorder.

**Figure 1:**
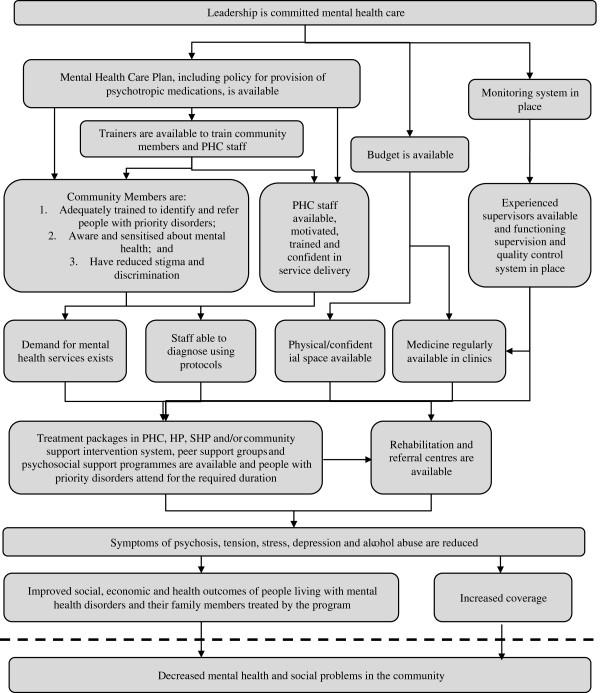
Theory of change.

After four consecutive workshops conducted alternatively with (mental) health care providers and policy makers, a final Theory of Change map was drawn up (see Figure 1). In the TOC methodology, only the final outcome was decided *a priori* as ‘improved social economic and health of people with mental disorders treated by the program’. The first workshop developed a broad outline of preconditions related to service delivery, problem identification and detection, mental health literacy and stigma reduction, capacity building, quality assurance mechanisms, budget availability and political buy-in. The TOC formulated at this stage included a pathway for outcomes and associated interventions within the health facilities that focus mainly on the capacity of health care workers to identify, treat and follow-up with patients. This pathway was combined with questions about the additional burden the proposed activities would pose on health staff. Another pathway was aimed at the community, which involved training community volunteers in identification, sensitization and providing support to people with mental health problems. Related to this, respondents emphasized the need to systematically include families, peer and schools in the process. The availability of community-level service delivery agents was seen as the most important hurdle. A third pathway within the overall TOC concerned outcomes related to the functioning of the health organization. These included the establishment of a supervision system and the policy for procurement of psychotropic medications. Respondents were particularly concerned about the feasibility of a regular and reliable drug supply chain. Fine-tuning of the TOC in subsequent workshops resulted in recommendations towards the inclusion of preliminary outcomes or interventions related to livelihoods, rehabilitation, referral mechanisms and technical oversight, as well as defining assumptions around the participation of service users and adopting an approach combining pharmacological treatment with community-based psychosocial support mechanisms.

The final TOC was the point of departure for the explorative qualitative interviews. Additional file 1 provides a summary of the results, organized as key barriers in the process of developing a mental health care plan and proposed solutions to overcome these.

A number of overlapping and crosscutting themes emerged from the qualitative data. These included the negative attitudes that surround mental health that are pervasive, that permeate all sections of society, and which are particularly difficult to tackle. Negative attitudes result in discrimination of people with mental health problems, and underlie problems related to detection, low demand and access to services and perceived feasibility of task-shifting. While increasing mental health literacy was a much-advocated strategy to overcome these attitudes, respondents were unequivocal that much depends on what information is imparted to whom. For instance, information about availability of services should be disseminated publicly, yet a better understanding of mental health problems should be addressed in more private spheres. Also, working with people that are generally trusted (e.g. community elders and traditional healers) in combination with people who are respected public figures (e.g. celebrities and political activists) would expectedly sort the largest impact. The situation presented was even more nuanced, as education and awareness alone were found to be insufficient to actually change attitudes, as that required actual involvement of key stakeholders with mental health issues.

A recurrent theme was that for a mental health care plan to be introduced there is an urgent need to improve access to services. Having treatment available in itself would not guarantee access and uptake of such services. Community members stated that it was a necessity that health workers were sensitive and maintained confidential when dealing with people with mental health problems. They also pointed towards a need to change the commonly held notion that services at the health posts are for the poor and that the more affluent should go to the district hospital. But also more practical concerns would need to be addressed in order to improve access. For instance, health workers reported that the detection of people with mental health problems is fraught with difficulties, in part due to the low mental health literacy and persistent negative attitudes, also and especially in the people closest to people with mental health problems. Still, many respondents believed that lay people, especially after receiving training, would be able to identify that something was wrong, even if not able to identify the type of mental health problems. However, in the absence of specialized resources in the community, respondents stressed that health workers are ultimately responsible for the detecting mental illness. Access can further be increased by more pragmatic measures that were proposed during several of the interviews by community members and health workers, such as arranging support for the recurrent transport to and from the health facilities. Efforts to improve identification and access need to be sensitive to unhelpful sentiments among both help seekers and health providers. Currently, the former feared accusations and discrimination and therefor hid their problems, while the latter feared anger and aggressivity from patients.

In many of the processes involved in the establishment of a mental health care plan the families of people with mental health problems play a crucial, albeit dual, role. Families appeared to be somewhat of a double edged sword. On the one end, families were seen as the key agents to help in improving the detection, access and maintenance of mental health treatment and care. On the other hand, family members, due to fear for loss of status or being discriminated against, take part in the ill-treatment or neglect that patients experience. For example the family members were reported to restrict access to care by protecting the client from stigmatization and hostility by hiding the illness; others were reported to threaten to reject him or her from the family all together.

Numerous issues were raised by the respondents with regards to making task-shifting feasible and acceptable. Beyond the obvious need for training, all respondents emphasized the risk of over-burdening health workers and the need for compensations for all those involved in task-shifting. The formal recognition of training and mandate of the work was for many health workers as important as financial reimbursement. According to the majority of respondents people in the community should also be trained in mental health care, rather than health workers alone. It was suggested to have focal points in each village, or for every 15–20 households, who can play a role in the care and support of people with mental health problems.

## Discussion

The literature on the integration of mental health into primary health care in LMIC is characterized by description of lessons learned from actual practice. To date, there is little formative research done in this area. The present study describes a systematic approach integrating formative research that provides data to inform the development of a comprehensive mental health care plan, incorporating the different perspectives of key stakeholders. We believe that a comprehensive methodology to identify and address barriers to acceptability and feasibility is important especially when applying mental health interventions cross-culturally [27]. The present combined-methods used in the formative research allows for the exploration of contextual knowledge of needed processes to put such plan into practice. In Nepal, a fragile state grappling with the aftermath of a decade of war and where even the most basic mental health services are unavailable in rural settings, this endeavor is met with many systemic obstacles. Health workers that are already overburdened, the unavailability of psychotropic medicines, and general unawareness of, or deeply engrained negative attitudes towards, people with mental health problems are some of the challenges introduced by stakeholders. At the same time however, developing mental health services anew also provides with opportunities to systematically build new structures [16]. The ability to plan for mental health services to take place concomitantly within the health facilities as well as the communities is a good example of this. The present study reveals the value of such a hybrid community-facility approach that includes a spectrum of interventions from tailor-made community sensitization and mobilizing community support to training health workers and a reliable supply of psychotropic drugs.

A strategy for decentralized mental health care has also been advocated in other post conflict settings such as Burundi [28],Uganda [29] and Lebanon [30]. While some initiatives towards a community mental health model have been implemented, or are presently ongoing, in Nepal [14,31], the government has not yet, policy notwithstanding, adopted a plan for the integration of mental health into primary health care.

As the systematic introduction of community mental health care cannot, from the onset, feasibly target all disorders, prioritization is required. Should governments choose to target the disorders that are most prevalent, those that cause most burden or those that can be addressed most feasibly? As such decision has potentially large implications for the country’s mental health strategy, it is important to have such prioritization done by key stakeholders in Nepal. The PRIME consortium has opted for depression disorder, alcohol use disorder and psychoses as priority disorders exactly because these impose the largest burden of disease and culturally acceptable interventions supported with robust evidence for effectiveness exists [17]. The results of the prioritization exercise, based on criteria of feasibility, acceptability and commonness, are largely congruent with those, except for the high priority given to epilepsy. The high importance given to epilepsy means that this will also be incorporated in the mental health care plan that is developed subsequent to this formative study.

The present study has a number of implications for the development of the mental health care plan in Nepal. The preliminary (i.e. before pilot-testing) care plan consists of different components. Within the community these included stigma reduction, sensitization, case detection, user group mobilization and focused psychosocial support. Within the health facility these included awareness raising, screening and assessment, pharmacological and psychosocial treatment, all of which are largely based on the mhGAP guidelines [9]. Training, drug-supply chain management, monitoring & evaluation and supervision are an integral part of the plan. The details of the care package will be described in an upcoming publication.

These components and interventions are informed by the present study in multiple ways. First, in the selection of priority disorders, in that epilepsy has been included based on the high priority score it received. Second, it has resulted in specific adaptations or strategies for implementation. Given the crucial and complex role of family members in the support for, and perpetuation of, mental health problems, interventions targeting families have been emphasized in the plan. Issues raised in relation to poor demand and access are addressed by a novel method for proactive case-finding by well-placed community-informants. Related, strategies aimed to reduce pervasive negative attitudes about mental health are now being developed encompassing the suggestions made in this study (including the sphere of intervention and most adequate delivery agent). Also, the concern about overburdening health staff and volunteers has led to a clear division of tasks and training of different cadres of health workers (i.e. making a distinction between prescribing and non-prescribing staff). Moreover, it has resulted in the decision to not rely on the cadre of community health volunteers (Female Community Health Volunteers). Even if appropriate, they are currently unpaid and are overburdened by multiple task-shifting roles. Finally, following recommendations by health workers in the study, to aid identification and detection at health facilities easy-to-use protocols and visual charts will be developed for frontline staff. Third, it has provided clear suggestions for additional foci in the plan. The need of a district-level focal point for mental health has since been advocated. Similarly, the need to formalize the training certificates now features on the agenda in discussions with the Ministry of Health. Together with the emphasis given on securing a reliable supply of pharmacological medicine, respondents warn for issues related to sustainability. The need to deal with the issue of sustainability right from the start has been one of the main lessons learned from other community mental health programs in LMIC [4]. The next phase of research within PRIME will be geared towards the evaluation of the strategies and plans that have been formulated as a result of the formative phase.

The process of the subsequent research steps feeding into each other has been important in formulating recommendations for practice. The priority setting defined the focus of the TOC, in turn the TOC defined the scope of the qualitative study. Furthermore, besides the concrete implications, by eliciting stakeholders’ opinions and their active involvement we hope that these methods will ensure ownership and buy-in on the development of a mental health care plan by stakeholders.

A few limitations should be noted. The research is conducted in the district where the further development and pilot-testing of the care package is going to take place. With the vast socio-economic, cultural and geographical differences, the findings may not necessarily generalize to all of Nepal. Second the priority setting exercise was largely based on purposive sampling. While we are confident that most senior mental health professionals were involved, the results are largely based on experts based in urban clinical settings. In addition, the low priority scores given to the developmental disorders are surprising, especially given other reports about their importance [14]. The scarcity of mental health professionals specialized in child and adolescence may have influenced these findings.

## Conclusion

Our research has laid out a comprehensive framework for setting priorities for mental health care in Nepal. It has outlined major challenges as well as recommending concrete strategies about how to overcome them. There was a strong endorsement of a hybrid system that encompasses community-, and facility-based care. Guaranteeing a sustainable supply of psychotropic medicine and making sure to not over-burden health workers or volunteers were identified as key challenges. The dual capacity of families, both the natural sphere of support for people with mental health problems and the ones maintaining or reinforcing negative attitudes towards sufferers, was also seen as needing attention. This study provides the foundation for further development and evaluation of integrated mental health care in Nepal.

## Competing interests

The authors declare that they have no competing interests.

## Authors’ contributions

MJ developed study design, was responsible for analyses, responsible for drafting and revising the manuscript; NL was responsible for implementation of the study, was involved in the development of the study design, contributed to drafting manuscript; MT was involved in the development of the study design, contributed to drafting manuscript; IK was involved in the development of the study design, contributed to drafting manuscript. All authors read and approved the final manuscript.

## Pre-publication history

The pre-publication history for this paper can be accessed here:

http://www.biomedcentral.com/1471-244X/13/332/prepub

## Supplementary Material

Additional file 1 **Highlight results from qualitative interviews.** This table provides an overview of the main findings from the qualitative study, presenting the results in terms of barriers and recommended solutions. Click here for file
