# Supplementary material for: Setting priorities for mental health care in Nepal: a formative study
Source: BMC Psychiatry. 2013 Dec 5;13:332. doi: 10.1186/1471-244X-13-332 (PMC4233621; doi:10.1186/1471-244X-13-332)
Supplement: Additional file 1 — Highlight results from qualitative interviews. This table provides an overview of the main findings from the qualitative study, presenting the results in terms of barriers and recommended solutions. [file 1471-244X-13-332-S1.pdf]

TOC	Barriers	Recommendations
Coordination and planning	**HMIS** program cannot be incorporated into District Level Health Information System until it is expanded nation-wide<br><br>**Lack of political buy-in for country-wide expansion** as mental health is not MDG, and due to lack of budget	**Coordinate a mutual plan with both ministries** to discuss policy on prescription of psychotropic drugs<br><br>**Increase Budget** from tobacco health tax and invest returns in mental health
Motivated and trained staff (task-shifting)	**Program acceptance.** Health workers can only dedicate time to mental health care if mandated by the government<br><br>**Shortage of human resources** due to posting policy and NGO staff recruitment resulting in frequent turnover<br><br>**Status of task sharing.** Health workers need financial compensation and opportunities for professional development. Also, they need to be mandated to prescribe medicines, refer clients and receive legal protection for malpractice<br><br>**Training** success depends on provision of allowances and incorporating exposure to patients	**Program integration** to make the plan and reporting part of existing government system<br><br>**Human resource** recommendations including appointing separate mental health staff and change positing policy<br><br>**For task sharing to work** compensation is essential. For lesser paid staff financial reimbursements are recommended, for others official (MoH) certification and legal mandate for the work can be considered.<br><br>**Training implementation** to be staggered to prevent staff shortage and to include onsite training at mental hospitals
Motivated community members	**Community health volunteers' work burden** as this cadre is over-used by multiple health initiatives; financial incentives are required	**Financial incentives for community health volunteers** to be arranged through a pension funds or for compensation-per-referral
Community	**Limited sensitivity** due to lack of mental health	**Activities and resources** include mass

identification	literacy and the immediate 'invisibility' of many symptoms<br><br>**Conflicting roles and responsibilities:** Family members are relied upon to detect problems, at the same time family members' reactions (i.e. imposed burden and shame) are a primary cause for hiding mental illness	sensitization(street dramas) about mental illness and training through community groups
Community awareness	**Lack of awareness** permeates *all* sections of society and castes (regardless of literacy level)<br><br>**Resistance to education.** Stigma reduces receptiveness to campaigns about mental illness.	**Strategies for awareness-raising:** Public awareness-raising for information about services and symptoms, and private strategies for providing deeper understanding.
Attitudes towards mental health	**Emotionally charged attitudes** including a combination of denial and hatred. Awareness of detrimental effect of stigmatization does not necessarily result in less collective negative emotional responses	**'Bottom-up' vs 'top-down' strategies** include grass-roots activity such as peer education, as well as laws and policy against discrimination.<br><br>**Reducing stigma;** reducing the dichotomy between treatments for the mind and body, and by using celebrities to promote treatment<br><br>**Beyond awareness-raising:** Awareness does not produce positive attitudes *per se*, it might increase negative emotions. In-depth education is needed alongside sensitization.
Demand for services	**Social and emotional factors** (humiliation, guilt, low self-esteem, shyness) accompany the social burden of seeking help.<br><br>**Unawareness** that health centers exist and a lack	**Improve confidentiality** by training health workers in trust-building with patients and protecting the family status. Confidential space for mental health consultations is needed.

	of clarity about whether treatments are cost free<br><br>**Poor reputation of services** due to lack of privacy and prevalence of abuse.	**Use mass media channels** to communicate information about services and create clarity on costs and available support.
Treatment package/ service delivery	**Consultations jeopardized** due to lack of gender matched consultants; lack of privacy; safety concerns due to fear of violent patients; lack of confidence in competencies of health staff<br><br>**Inadequate infrastructure** is characterized by a poor referral system, resources and options.	**Control distribution** of psychotropic medications to prevent drug abuse<br><br>**Improved referrals** by having psychiatrists attending training to meet health workers that will refer to them; and by creating additional 'mental health beds' in existing hospitals
Recovery and treatment adherence	**Stigma, discrimination and abuse** cause exclusion of mentally ill people from social functions, religious rituals, and income generating organizations, which negatively influences their chances of recovery<br><br>**Patients drop out of treatment due to** (a) lack of money or family support for treatment or transportation; (b) offensive behavior of health workers; (c) received treatment does not meet expectations, and (d) being talked into traditional healing<br><br>**Patients discontinue medications** due to associated cost, side effects, (absence of) symptom reduction, as well as beliefs that medicine use is indefinite and makes one weak	**Raise Awareness** that mental illness is treatable through community sensitization<br><br>**Improve social standing of clients** through vocational training and income generating<br><br>**Re-integrate clients into community** by engaging them in community activities using volunteers.<br><br>**Treatment adherence can be improved** by providing treatment free of charge and by involving community and peer groups for motivation, education, and frequent follow-up and home visits. **Build trust with health care workers** by maintaining confidentiality and providing sensitive & quality care
Supervision and quality	**Under qualified supervisors** are not taken seriously by service providers	**Create a post for District Mental Health Coordinator** to prevent negligence of, and

control	**Supervision is irregular or non-existent,** in part due to lack of incentives<br><br>**Threats, disrespect, abuse and punishment** of health workers during supervision	assure funds for, supervision, as well as monitor the supervisors(observation of trained health workers, visiting patients, reviewing records)<br><br>**Quality Control** can be ensured by having coordinator and supervisors report outcomes.
Family involvement	**Ambivalence** among family member, as many know that giving support is needed, yet feeling hostile towards dysfunctional family members<br><br>**Lack of support for married women** due to actual or feared rejection from in-law families if mental illness is revealed	**Protecting family status** by support services protecting the family (as well as the patient) from stigmatization. The influence of heads of families should be used to encourage help-seeking
Community support	**Lack of social support for access,** as clients fail to seek or maintain treatment if accompaniment and transportation is unavailable	**Community members or friends** play an essential role by accompanying help seekers, including, but not limited to, arranging logistics
Drug procurement	**Medication not on the essential drug list** causes concerns over budget, maintenance of government supply and expired medicines	**Alternatives to the essential drug list** include health facilities partnering with local pharmacies to purchase and stock psychotropic drugs
Referral care	**Potential for confusion and stress** if advice from health centers contradicts traditional healers<br><br>**Lack of quality from hospitals,** despite being perceived as more desirable than health posts	**Referrals from traditional healers** are an essential pathway to care for the poor, through referral to health facilities

Note: MoH Ministry of Health; TOC Theory of Change; HMIS Health Management Information System; MDG Millennium Development Goals
